# Development and initial evaluation of a smartphone application based on acceptance and commitment therapy

**DOI:** 10.1186/2193-1801-1-11

**Published:** 2012-07-31

**Authors:** Kien Hoa Ly, JoAnne Dahl, Per Carlbring, Gerhard Andersson

**Affiliations:** 1 Department of Behavioural Sciences and Learning, Linköping University, Campus Valla, 581 83 Linköping, Sweden; 2 Department of Psychology, Uppsala University, von Kraemers allé 1A-C, 751 42 Uppsala, Sweden; 3 Department of Psychology, Umeå University, Beteendevetarhuset, 901 87 Umeå, Sweden; 4 Department of Clinical Neuroscience, Division of Psychiatry, KarolinskaInstitutet, Karolinska University Hospital Huddinge, 141 86 Stockholm, Sweden

## Abstract

**Background:**

An intervention, consisting of an ACT-based smartphone-application and a web-based psychoeducation, has been developed. The smartphone-application, together with the psychoeducation, aims to function as a self-help intervention for living consistently with one's values. The study is an exploratory investigation of this new smartphone-based tool.

**Case description:**

Primarily, the study aims at investigating a new field, providing a basis for generating hypotheses for further research. The first aim of this initial, exploratory study was to examine if this intervention had an effect on the variables of: valued actions, psychological flexibility, and life satisfaction as well as the states of depression, anxiety and stress, for a non-clinical sample of 11 Swedish Iphone users. This was made with a quasi-experimental pretest-posttest design without control group. The second aim was to investigate how the participants experienced the intervention, as measured by a qualitative questionnaire.

**Discussion and evaluation:**

The group analyses showed that the participants increased their valued action and psychological flexibility significantly during the intervention. Furthermore, value-based actions and psychological flexibility showed small effect sizes when comparing pretest and posttest score. However, the design of the study makes it impossible to draw any certain conclusions. The qualitative questionnaire showed a general positive experience of the intervention.

**Conclusions:**

The results from the present study indicated that the intervention should be studied further. The findings also generated a number of hypotheses that could be investigated in further research.

**Electronic supplementary material:**

The online version of this article (doi:10.1186/2193-1801-1-11) contains supplementary material, which is available to authorized users.

Acceptance and Commitment Therapy (ACT) has shown to impact a wide variety of mental health problems including depression, work-site stress, and a variety of anxiety disorders ([Strosahl et al., 2004]). ACT has also affected behavioral medicine areas important to public health including obesity, chronic pain, smoking, and diabetes self-management ([Strosahl et al., 2004]). The ultimate goal of ACT is to help a client gain a sense of life direction that is consistent with his or her values and then begin acting in a way that is in line with those values ([Strosahl et al., 2004]). The use of new mobile technologies (e.g. smartphones) may be useful in facilitating ACT’s goal due to its popularized accessibility into mainstream culture. Mobile terminals have become an important part of our everyday lives, and they are already providing us with several services. Therefore, adding applications into new mobile technologies may be a natural step to facilitate healthier living ([Tufano & Karras, 2005]). [Boschen and Casey (2008]) summarized the advantages of using mobile phones in cognitive-behavioural therapy (CBT), and the same advantages apply to smartphones: 1) mobile, 2) accepted in society in general, 3) relatively cheap (this also applies to smartphones which on average tend to be less expensive than computers), 4) they are a device with comparatively low on-going maintenance costs, 5) they are a device already owned by a large number of people, 6) they are almost always on in that they continue to operate, 7) almost always connected, 8) they are programmable, meaning they are able to run novel applications software, 9) they are capable of recording media, including audio, photographs, and in many cases video, as well as being able to play or show these media to the user, 10) they are capable of interacting with the user to allow input of data using a keypad, keyboard, or touch screen, and 11) and they are generally designed to be easy to use for most of the population.

The advantages of mobile phones have also been contrasted to Internet-delivered interventions. Although the Internet is effectively used for distributing psychological treatment programs to improve mental health ([Andersson, 2009]; [Barak et al. 2009]; [Andrews et al. 2010]), it has been argued that Internet-based treatment applications have limitations ([Andersson & Cuijpers, 2008]) ([Andersson, 2010]). One important limitation is the lack of assimilation into the user’s daily life, which is a crucial aspect of CBT-based treatments ([Martell et al. 2010]). Even if paper versions of text material can be used, this option may not be practically feasible. Mobile phones, on the other hand, are ubiquitous and hence can be present all the time. Nowadays, access to Internet can be facilitated by means of smartphones. [Mattila et al. (2008]) also argue that the weaknesses of Internet-based wellness management tools could be the lack of assimilation into the user’s daily life. They believe that the need to have a computer and Internet connection to access a wellness website makes wellness management a separate task, which needs to be allocated a time slot rather than an integral part of life. Mattila et al. state that smartphones offer potential for providing wellness management tools due to its accessibility and, independence of time and location.

Another limitation of Internet-based treatments for psychological disorders, delivered via computers, is that they tend to be text-based, often reaching close to 200 pages of reading ([Bendelin et al. 2011]). Smartphone applications tend to be more user-friendly and condensed than regular web pages (for example the difference between a smartphone application for a newspaper and web pages for the same newspaper), which could be an attractive feature for persons with low-literacy skills, who need encouragement to work through the program ([Andersson et al. 2007]). The encouraging aspect is important, as guidance appear to be crucial in order to prevent dropout from guided self-help treatments ([Christensen et al. 2006a2006b]), which was recently confirmed in a meta-analysis ([Cuijpers et al. 2010]). The current intervention is based on our previous experiences with Internet-based depression treatment ([Cuijpers et al. 2010]; [Andersson et al. 2011]; [Andersson et al. 2005]; [Bendelin et al. 2011]; [Holländare et al. 2011]; [Meyer et al. 2009]; [Vernmark et al. 2010]). Thus, the concept of the intervention was built with the intention to target some of the aspects of current Internet-delivered interventions that could be improved.

For the purpose of the current paper an intervention, consisting of an ACT-based smartphone-application and a web-based psychoeducation, has been developed. The smartphone-application, together with the psychoeducation, aims to function as a self-help intervention for living consistently with one’s values. It is suggested that the degree to which a person lives up to his or her values will directly affect that person’s sense of quality of life ([Lundgren et al., 2012]). A correlation analysis shows that higher perceived value-action discrepancy is positively correlated with measures of psychological distress, using the Depression, Anxiety and Stress Scale (DASS-21; [Lundgren et al., 2012]), Furthermore, higher value action discrepancy is associated with lowered psychological flexibility, as measured by the Acceptance and Action Questionnaire II (AAQ-II), and lower levels of subjective well being, as measured by the Satisfaction With Life Scale (SWLS; [Lundgren et al., 2012]). Thus, living a life that is in line with one’s value appears to improve life satisfaction and psychological flexibility, as well as emotional states of depression, anxiety and stress.

The study is an exploratory investigation of this new smartphone-based tool. Primarily, the study aims at investigating a new field, providing a basis for generating hypotheses for further research. The intervention was designed to focus on two of the six core processes in ACT, namely values and committed actions ([Hayes et al. 1999]). According to [Strosahl et al. (2004]), the core processes of values and committed actions are regarded as the main purposes of ACT. The intervention aims to be flexible and useful for non-clinical populations and therefore does not target any specific syndrome. In the present study, ACT was chosen as a theoretical framework due to its emphasis on living a value consistent life, rather than symptom reduction. It can be argued that there is a need for easy and accessible psychological interventions for individuals with minor levels of psychological distress and without a diagnosis.

The first aim of this exploratory study was to examine if the smartphone application together with the psychoeducation would have an effect on measures of valued actions, psychological flexibility, and global satisfaction as well as the symptoms of depression, anxiety and stress. The second aim was to investigate how the participants experienced the intervention. An open-ended questionnaire was used to examine how the participants experienced the content of the intervention, as well as the intervention as a mobile-based platform. We also examined when, where and how often the participants used the application.

## Method

### Participants

Participants were 11 volunteers (4 females and 7 males; mean age 29.5 years, SD = 5.96, range from 22 to 42 years old), recruited from a website, that writes about popular scientific psychology. In order to be included, participants had to be at least 18 years of age and have continuous access to an Iphone. Since the study is an exploratory investigation of this new field, the participants were not allowed to suffer from any major psychiatric condition or undergoing treatment. Nevertheless, in the pretest, one participant showed slightly elevated levels of self-reported depression and anxiety, as measured with the short version of the Depression Anxiety Stress Scale (DASS-21; [Lovibond & Lovibond, 1995]). Except from provision of the Iphone application, no inducement for participation was offered.

### Measures

#### Valued actions

Valued actions were evaluated using the Bull’s Eye Value Survey (BEVS; [Lundgren, et al., 2012]). BEVS uses a 7-point scale and is sensitive to treatment effects and can differentiate between clients who receive values-based interventions and those that do not ([Lundgren, et al., 2012]). BEVS uses the visual representation of a dartboard and respondents are asked to mark an “X” somewhere on the target to signify the extent to which their current behaviors are consistent or inconsistent with the stated value in the four life domains; relationships, leisure, work/education and personal growth/health. The test-retest reliability coefficient for the total BEVS value action discrepancy score is *r* = .85. The test-retest correlation coefficients for the four domains are: Relationships *r* = .80, Leisure *r* = .74, Work/Education *r* = .81, Personal growth/Health *r* = .77. The test-retest correlation coefficient for believability is *r* = .94 ([Lundgren et al., 2012]).

#### Psychological flexibility

The Acceptance and Action Questionnaire II (AAQ-II) is a 10-item, 7-point scale that assesses psychological flexibility – a term that is used to describe the ability to accept unwanted internal experiences and engage in valued actions ([Hayes et al. 2004a]). Respondents rate the degree to which they agree or disagree with each statement on a 1 (never true for me) to 7-point (always true for me) scale. AAQ II has shown good reliability (test-retest reliability = .81-.87) and validity ([Hayes et al. 2004b]).

#### Global satisfaction

The Satisfaction With Life Scale (SWLS) is a 5-item self-report, 7-point scale that measures global satisfaction ([Diener et al. 1985]). SWLS has shown strong internal reliability (Cronbach’s Alpha = .87) and good temporal stability (test-retest correlation *r* = .82). The SWLS has also been shown to correlate with various measures of subjective wellbeing (*r* = .50).

#### States of depression, anxiety and stress

The Depression, Anxiety and Stress Scale (DASS-21) is a short version of DASS-42 and is a widely used scale to measure depression and anxiety in clinical research ([Lovibond & Lovibond, 1995]). DASS is a 21-item, 4-point scale and has demonstrated excellent psychometric properties, with Cronbach’s Alpha of .88 for the depression scale, .82 for the anxiety scale, .90 for the stress scale and, .93 for the total scale. DASS-21 has also shown to be valid ([Brown et al. 1997]).

#### Experiences of the intervention

A questionnaire containing seven questions, was created and distributed together with the posttest, to evaluate how the participants experienced the intervention. The questionnaire contained four open-ended and three closed-ended questions along with follow-up questions. A questionnaire survey with open-ended questions can be used as a means to understand and improve medical care services from the point of view of the health care consumer ([Bankauskaite & Saarelma, 2003]).

### Procedure

Initial recruitment was conducted through an invitational blog-post via a website, that writes about popular scientific psychology. Following recruitment, participants were distributed the pretest measurements, including BEVS, AAQ-II, SWLS and DASS-21, which were completed online. Individual emails with coded links were sent to participants to complete the questionnaires, which could be conducted either via mobile phone or computer. One week after pretest measurements had been completed participants were sent an email guiding them to install the file for the application and complete the psychoeducation. Following the psychoeducation, participants were instructed to use the application as much as they wanted for four weeks after which, the posttest would be sent to them. Posttest measurements, together with the questionnaire on experiences of the intervention, were sent out to the participants on the fourth week after the application had been sent out.

### Intervention

The intervention consisted of a smartphone application and a web-based psychoeducation.

#### Psychoeducation

The psychoeducation is intended to be easy and simple, with only the most essential content to get the application started. Therefore, the education focused only on the core processes of values and committed actions. The psychoeducation was presented on the web and thus could be viewed either on the mobile phone or on a computer. This education consisted of eight pages (a total of 3,714 words), presented in four modules. In addition to text-material, the education consisted of pictures, animations, cartoons, charts and short videos that aimed at illustrating the principles of the intervention. The participants were told to reserve about 45 minutes for the psychoeducation.

The first module introduces the participant to the present study and the theory of ACT, focusing especially on the core processes values and committed actions ([Wilson & Murrell, 2004]). The second module, *How to use values as a tool for behavioral activation*, is the main part of the education and starts with a behavioral experiment with the aim to teach the participant how values can be expressed as concrete behaviors. This module includes text-material that is inspired by a self-help book on ACT ([Hayes & Smith, 2005]), as well as complimentary illustrative videos. The third module, *Finding your values*, guides the participant to complete exercises defining his or her own values. The first exercise is inspired by the funeral exercise (see [Hayes & Smith, 2005], p. 166–170) with the exception that the funeral is changed to a centenary party. Another exercise helps the participant organizing his or her defined values into major life domains. The participant is guided in the use of life domains as they are defined in the BEVS: work/education, relationships, leisure, health/personal growth ([Lundgren et al., 2012]). A final exercise aims at helping the participant to break down the defined values into concrete behaviors. The final module is a detailed description on how to install and start the application as well as how to add life domains, values and behaviors into the application Participants are encouraged to use the application freely during the study. This is to prevent the application from becoming too demanding and thus lower the participants’ motivation to use the application.

#### Smartphone application

The main functionality of the smartphone application is to make it easy for the user to remember and register behaviors that are in line with their values. The participants are able to check off whenever they complete a behavior. Statistics of qualitative data (behavior frequency) is presented in the mobile application, so the participant can track their own progress.

**Figure 1:**
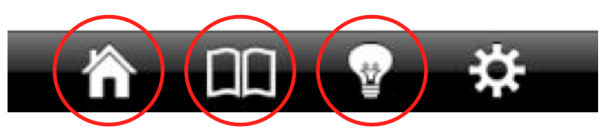
**The application’s menu.** The three main pages: Home, Book and Light Bulb marked with red circles. The fourth page, Settings, was disabled for the present study.

**Figure 2:**
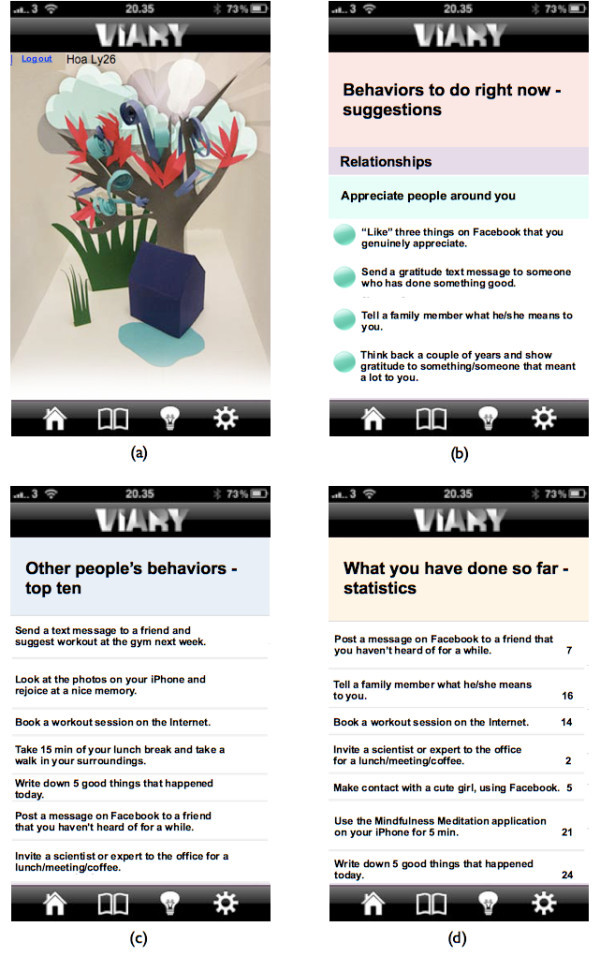
**Four screenshots from the application.** Picture **(a)** shows the main page for the Home page. Picture **(b)** shows suggestions from the user’s list of behaviors. Picture **(c)** shows top ten of other people’s recent behavior. Picture **(d)** shows statistics for what behaviors and how many times the individual user has done them so far.

The application contained three main pages: Home, Book and Light Bulb. The last page, Settings, is disabled for the present study. Figure 1 shows the icons of the three main pages. When clicking on the Home icon, picture (a) in Figure 2 is shown. The Home page displays the following key functions: suggestions from the user’s list of behaviors (picture (b) in Figure 2), top ten of other people’s recent behavior, (picture (c) in Figure 2), and statistics, showing what behaviors and how many times they have been done by the individual user so far (picture (d) in Figure 2).

**Figure 3:**
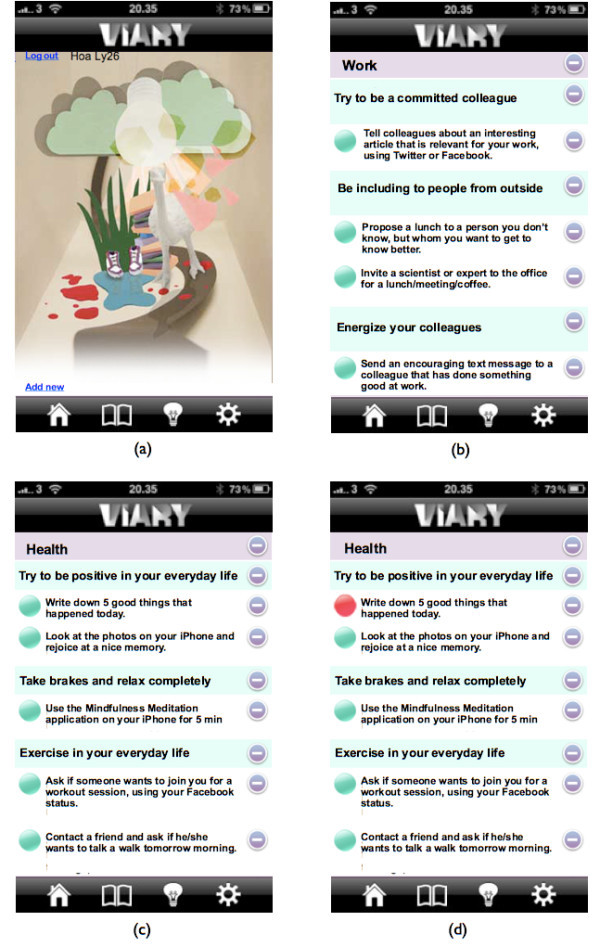
**Four screenshots from the application.** Picture **(a)** shows the main page for the Book page. Picture **(b)** and **(c)** show the database for the life domains: Work and Health, and the values and behaviors that appertain to them. Picture **(d)** shows a behavior that has been checked off.

When clicking on the Book icon, picture (a) in Figure 3 is shown. On the Book page, the user is able to add new values and behaviors into their application. The Book page also displays the following key function: the whole list of life domains (grey field), the whole list of values (green field) and the whole list of behaviors (white field), as seen on the examples on picture (b) and (c) in Figure 3. This page is the user’s own database of life domains, values and behaviors. The green buttons to the left of each behavior are checking off-buttons. When pressing one of these buttons, which the user was told to do after a behavior had been completed, the green button turned into a red button as shown on picture (d) in Figure 3. When the button turned red, the behavior was automatically registered in the statistics on the Home page (picture (d) in Figure 2). A button stays red for 24 hours before it turns green again. During this time, the user is not able to check off the same behavior again. This is to avoid users from checking off only one, or a few behaviors multiple times and thus motivating the user to try new behaviors from the database. In addition, the user is able to delete life domains, values and behaviors from this page by clicking on the grey circle with the white horizontal line on the far right. In order to differentiate the application from a to-do list, participants were told that it was important to delete behaviors, as well as values, from their application if they did not feel motivated to accomplish them.

**Figure 4:**
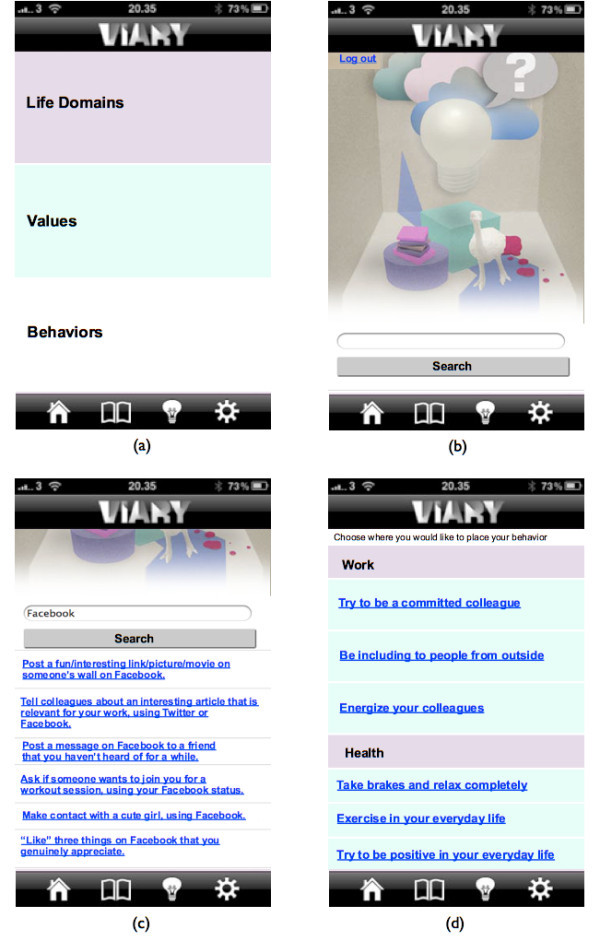
**Four screenshots from the application.** Picture **(a**) shows the main page for the Light bulb page. Picture **(b)** shows the search page. Picture **(c)** shows how to search in the open database. Picture **(d**) shows how to add a behavior, directly into the application from the open database.

When clicking on the Light bulb icon, picture (a) in Figure 4 is shown. On the Light bulb page, the user is able to search in the open common database. When clicking on either life domains (grey field), values (green field) or behaviors (white field), the user is sent to a search page as shown on picture (b) in Figure 4. The user is able to search on a certain word, e.g. Facebook, as seen on picture (c) in Figure 4, and when doing this, the user is able to see other people’s behavior that had to do with Facebook. In that way, users could inspire and get inspired by each other. When clicking on one of the behaviors, user is able to add the behavior directly into their own application (picture (d) in Figure 3).

The main function of the application is to define values within different life domains. These values are subsequently operationalized into concrete behaviors that can be performed directly. The user starts by defining both values and concrete behaviors in the application. For example: the value “Appreciate friends and family”, can be transformed to a concrete behavior that can be performed directly as: “Send a text message to someone that you care about, who has done something for you lately”. Values and behaviors in the application are not fixed, but can be flexible and adjustable by the individual user. Thus, users have the possibility to fill the application with individually tailored content. The purpose of this feature is, in line with previous research ([Christensen et al. 2006a]; [Strecher, 2007]), to increase adherence and user satisfaction. All behaviors that are typed in by the individual user can be automatically sent to an open database anonymously, which makes it possible to share behaviors among other users. Participants are informed about this possibility during the psychoeducation. The purpose of this feature is to make it possible for users to view other users’ values and behaviors, and thus become aware that other people are working with their values as well. Also, this feature functions as a source of inspiration, as the user can add behaviors that other users have contributed to the database. Social support can be an important factor to increase adherence and motivate users to continue an intervention ([Christensen et al. 2006b]; [Bell, 2007]; [Consolvo et al., 2006]; [Toscos et al., 2006]). The feature of the database makes it possible to view other users’ values and behaviors and intends to function as a social support in the application.

### Data analysis

The quantitative analysis was done using Statstica 6.0. Index on dependent variables, including SWLS, AAQ-II, BEVS and DASS-21, was analyzed using dependent *t*-test on pre to post changes in score. In addiction, Cohen’s *d* effect sizes were calculated using Excel. The qualitative data contained answers from 11 participants. No analysis was done. All answers from the participants were presented in the results.

## Results

### Group analysis

Table 1 shows the obtained data for the outcome measures. Paired samples *t*-test showed significant differences between pre and post intervention for the BEVS (valued actions), and AAQ-II (psychological flexibility). No significant differences were found on the SWLS (global life satisfaction), or on the DASS-21 (depressive and anxious symptoms). Cohen’s *d* effect sizes, adjusted with Öst’s (2006) recommended limit of value for within-group analysis (0.50 for small effect, 0.80 for medium effect, and 1.10 for large effect) show small effect sizes between pre and post intervention for valued actions, measured by BEVS and psychological flexibility, measured by AAQ-II. No meaningful effect sizes were obtained for the other measures.

**Table 1:** **Outcome measures**

	M (SD) Pretest	M (SD) posttest	Paired t-test^a^	Effect sizes^b^
EVS	17.73 (3.35)	19.91 (2.51)	t_(10)_ = −2.87*	d = −0.77
AQ-II	50.18 (9.90)	54.27 (7.16)	t_(10)_ = −3.06*	d = −0.50
WLS	24.18 (4.85)	24.73 (5.57)	t_(10)_ = −0.42 NS	d = −0.11
DASS-d	4.09 (6.04)	2.45 (2.11)	t_(10)_ = 1.15 NS	d = 0.38
ASS-a	2.45 (5.56)	1.36 (2.29)	t_(10)_ = 1.08 NS	d = 0.27
ASS-s	6.09 (4.18)	5.18 (2.86)	t_(10)_ = 0.78 NS	d = 0.27

*Note.* N = 11; BEVS = Bull’s Eye Value Survey; AAQ-II = Acceptance and Action Questionnaire II; SWLS = Satisfaction With Life Scale; DASS-d = Depressions subscale in Depression, Anxiety and Stress Scale; DASS-a = Anxiety subscale in Depression, Anxiety and Stress Scale; DASS-s = Stress subscale in Depression, Anxiety and Stress Scale.

^a^ = Dependent *t*-test.

^b^ = Cohen’s d.

*p <.05, **p <.01, ***p <.001, Ns = Not significant.

### Individual analysis

Information obtained from the qualitative questionnaire is presented for each subject in the Additional file 1. In general, the participants reported that the intervention made them think of their values and behaviors more. The majority of the participants used the application a couple of times per week and some of the participants were using it when sitting on the bus, metro, train etc.

## Discussion

The current intervention consisted of a smartphone application and a web-based psychoeducation. The intervention aimed to target the variables of valued actions, psychological flexibility, global satisfaction, as well as states of depression, anxiety and stress, using the ACT principles values and committed actions. The design of the study makes it impossible to draw the conclusion that the results are attributable to the intervention itself. The group analyses show that participants significantly increased their valued action, measured with BEVS during the intervention. In addition, participants increased their psychological flexibility, measured with AAQ-II significantly when comparing pretest and posttest measurement. Furthermore, value-based actions and psychological flexibility showed small effect sizes when comparing pretest and posttest score. The dependent variables of global satisfaction, measured by SWLS, and states of depression, anxiety and stress, measured by DASS-21, showed neither significant differences, nor effect sizes. However, these measurements might have been insensible for the population in the current study, and floor effects might have occurred. More rigorous studies are needed to establish if the current treatment approach is effective.

The current paper points at some features that could be tested in controlled studies. For example, it may be that the smartphone application could be used to increase adherence ([Boschen & Casey, 2008]), which is a problem in many treatment settings including Internet-delivered CBT ([Andersson, 2009]). We note other implications of our preliminary findings.

First, the intervention was mainly based on the platform of a smartphone. Smartphones might help to assimilate the treatment into the users’ daily life in a better way ([Mattila, et al., 2008]). The present study gives some support for that notion as participant 3 used the applications in situations where it was difficult to use a computer connected to the Internet, for example on the bus. The comment made by subject 1 saying: “simply seeing the icon of the application on the phone made me think of my values and behaviors more”, point to another advantage of using mobile phones as platform for health interventions. More generally, this suggests that smartphone applications may increase awareness of being in treatment in everyday settings. This does not necessarily have to be a good thing, but might help clients who need reminders to engage in homework and other treatment related activities outside of the therapy room. It is our hope that our preliminary findings will motivate researchers to continue developing health care applications for mobile phones (including modern smartphones), both for self-help interventions and as adjunct tools in face-to-face treatments. Other research groups have already started to develop these kinds of tools (e.g. [Bockting et al., 2011]).

Second, the open database of examples from clients that can be used for advice when generating valued activities is another unique feature of the current study. This utilized the social support that can be derived from other users of the application in an incremental manner, but raises some privacy issues. The outcome from the open-ended questionnaire showed that participant 2 experienced some difficulties with the open database, where the other participants’ values and behaviors were gathered. Also, participant 2 reported that if the open database would have been filled with more examples from the beginning, it might have reduced the demanding startup phase, and thus been a factor that could have motivated the participant to use the application more. Currently, most Internet administered psychological interventions do not involve implicit participant interactions except on forums. The way that the application creates social support, inspired by Consolvo and colleagues’ (2006) mobile application for encouraging physical activity where information can be shared among other users, has not been fully tested yet, but could be a field for further research.

Third, the individually tailored material of the application seemed to increase adherence to the intervention. For example, participant 1 reported that she started the application when she came up with a new value or a new behavior. This suggests that the possibility to add individual material to the application leads to more use. Participant 6 accounted for erasing and adding behaviors during the whole study. The participant reports that this flexibility of the application was a good feature. This might be a promising field for further research. The current intervention has highlighted a cost-effective way of providing individually tailored material since the users of the application create the individual material themselves.

Fourth, the current study investigates an intervention that focuses little on text-based material and more on other media, like movies, pictures, cartoons and animations, and most of all; behavioral experiments and exercises. Findings imply that it might be important to find other ways to motivate patients to complete self-help interventions, for example two participants reported that the cartoons and the movies made the psychoeducation more pleasurable. Nevertheless, three participants had the opinion that the psychoeducation was too long. Thus, despite that the psychoeducation was constructed to be short and easy, containing eight pages, three out of eleven participants experienced the education to be too demanding. These findings indicate what previous research already has highlighted: that text-materials in self-help treatment can be perceived to be too demanding by patients. Also, this indicates that an integration of different media, like pictures, animations, movies and sound clips can reduce the demanding material and make the psychoeducation more pedagogic.

This implication becomes even more critical for the low educated population. A study by [Birru et al. (2004]) indicates that the low-literacy adult population faces difficulties when using the internet to find health-related information. Simultaneously, according to World Health Organization (WHO, n.d.), one determinant for health is education; low education levels are linked with poor health, more stress and lower self-confidence. The development of technology, giving developers the possibility to integrate different media outlets including movies, animations, sound clips, pictures, cartoons, etc. in a more advanced manner, could be a way to facilitate more pedagogic self-help interventions. Further research is needed to be able to develop self-help interventions that are effective for a more broad population. Integrating social media sites, for example, might be helpful for this purpose. Social media sites that many people already use in their daily life could be a bridge to reach people that otherwise would not seek information about mental health problems.

### Limitations of the present study

There are a number of limitations of the study that make it difficult to generalize the results. First, we used a non-clinical convenience sample of volunteer participants. Second, the study is limited by a lack of a control group, which makes it difficult to draw the conclusion that the intervention itself was the factor that caused the improvement of the dependent variables of valued actions and psychological flexibility; it is for example difficult to exclude history as a confounding variable. Furthermore, it is difficult to evaluate if the findings are attributable to the smartphone application itself, to the psychoeducation itself, or to the whole intervention. However, the current paper was an initial, exploratory study with the aim to investigate the application and generate hypotheses rather than to be a traditionally complete RCT study to determine the exact effects of the intervention.

The exploratory nature of the study motivates the use of a qualitative questionnaire. Nevertheless, in-depth interviews with the participants might have been a better alternative for exploring their experience and opinion of the intervention. A questionnaire with set questions faces the risk of being too leading. The study can, despite the small sample and attributes that make it impossible to exclude threats to the external and internal validity, give information about participants’ opinion and experience of the intervention. Learning more about the difficulties as well as the positive aspects that each participant experienced during the study can yield important directions for future development of the intervention.

## Conclusions

Finding ways to develop accessible and cost-effective psychological interventions is important as it is unlikely that clinicians will be able to serve all clients in need with traditional face-to-face interventions. Mobile technologies have the potential to facilitate this work. Thus, there is reason to believe that a substantial part of Internet-based interventions in the future will be executed through smartphones or at least supported by smartphones.

## Electronic supplementary material

Additional file 1: **Appendix.** Subject 1 is a 42-year-old man. He reports that he has used the application a couple of times per week. Also, he reports that he started the application mostly when he was at home. His general experience of the application is that the content is good, although difficulties with handiness made the application hard to use. The psychoeducation, he reports, was basic and good. The subject accounts for being more aware of his own values and behaviors after starting to use the application. He reports that he has accomplished more good behaviors from his behavior repertoire during the study. Table 3 shows pre and posttest data for subject 1 from the quantitative measurements. (DOC 721 KB)
