# Supplementary material for: Development and initial evaluation of a smartphone application based on acceptance and commitment therapy
Source: Springerplus. 2012 Jul 31;1:11. doi: 10.1186/2193-1801-1-11 (PMC3725853; doi:10.1186/2193-1801-1-11)
Supplement: Supplementary file 1 — Additional file 1: Appendix. Subject 1 is a 42-year-old man. He reports that he has used the application a couple of times per week. Also, he reports that he started the application mostly when he was at home. His general experience of the application is that the content is good, although difficulties with handiness made the application hard to use. The psychoeducation, he reports, was basic and good. The subject accounts for being more aware of his own values and behaviors after starting to use the application. He reports that he has accomplished more good behaviors from his behavior repertoire during the study. Table 3 shows pre and posttest data for subject 1 from the quantitative measurements. (DOC 721 KB) [file 40064_2012_9_MOESM1_ESM.doc]

# **Appendix**

*Subject 1.* Subject 1 is a 42-year-old man. He reports that he has used the application a couple of times per week. Also, he reports that he started the application mostly when he was at home. His general experience of the application is that the content is good, although difficulties with handiness made the application hard to use. The psychoeducation, he reports, was basic and good. The subject accounts for being more aware of his own values and behaviors after starting to use the application. He reports that he has accomplished more good behaviors from his behavior repertoire during the study. Table 3 shows pre and posttest data for subject 1 from the quantitative measurements.

Table 2

*Answers from subject 1 on the qualitative questionnaire, translated into English.*

Table 3

*Raw data on the measurements of BEVS, AAQ-II, SWLS, DASS-21-d, DASS-21-a and DASS-21-s for subject 1.*

|  | **Pretest** | **Posttest** |
| --- | --- | --- |
| **BEVS** | 18 | 20 |
| **AAQ-II** | 53 | 55 |
| **SWLS** | 26 | 31 |
| **DASS-21 - d** | 1 | 1 |
| **DASS-21 - a** | 1 | 0 |
| **DASS-21 - s** | 3 | 2 |

*Note.* N = 1; BEVS = Bull’s Eye Value Survey; AAQ-II = Acceptance and Action Questionnaire II; SWLS = Satisfaction With Life Scale; DASS-d =Depressions subscale in Depression, Anxiety and Stress Scale; DASS-a =Anxiety subscale in Depression, Anxiety and Stress Scale; DASS-s = Stress subscale in Depression, Anxiety and Stress Scale.

*Subject 2.* Subject 2 is a 30-year-old man. He reports that he has used the application once during the whole study. He reports that he started the application at home only when he installed the application. His general experience of the application is that the startup phase was too long and demanding, which made him use the application only once during the study. If it would have been easier to overview the open database with other participants’ values and behaviors in the application, or if the application would have been filled with examples from the beginning, it might have made it easier to use the application more, he reports. The psychoeducation, he reports, was informative, as well as easy to understand, and the cartoons and pictures made the education more pleasurable. The subject accounts for being more aware of his own values and behaviors after starting to use the application, as well as trying to complete new behaviors. He reports that, because of the psychoeducation and The application, he realizes the importance of small behaviors, and that living in line with one’s values does not have to be as difficult as first thought. Table 5 shows pre and posttest data for subject 2 from the quantitative measurements.

Table 4

*Answers from subject 2 on the qualitative questionnaire, translated into English.*

Table 5

*Raw data on the measurements of BEVS, AAQ-II, SWLS, DASS-21-d, DASS-21-a and DASS-21-s for subject 2.*

|  | **Pretest** | **Posttest** |
| --- | --- | --- |
| **BEVS - total** | 14 | 18 |
| **AAQ-II** | 51 | 55 |
| **SWLS** | 25 | 29 |
| **DASS-21 - d** | 8 | 1 |
| **DASS-21 - a** | 1 | 1 |
| **DASS-21 - s** | 11 | 3 |

*Note.* N = 1; BEVS = Bull’s Eye Value Survey; AAQ-II = Acceptance and Action Questionnaire II; SWLS = Satisfaction With Life Scale; DASS-d =Depressions subscale in Depression, Anxiety and Stress Scale; DASS-a =Anxiety subscale in Depression, Anxiety and Stress Scale; DASS-s = Stress subscale in Depression, Anxiety and Stress Scale.

*Subject 3.* Subject 3 is a 25-year-old woman. She reports that she has used the application every other or every third day during the study. Also, she reports that she started the application mostly at night before going to bed. Her general opinion is that the application was easy to use and that it felt satisfying whenever a behavior from the database was completed and checked off in the application. The psychoeducation, she reports, was easy to understand, and was neither too long, nor too short. The subject accounts for being more aware of her own values and behaviors after starting to use the application, as well as trying to complete new behaviors. She reports that some kind of therapeutic support would have motivated her to use the application more. Table 7 shows pre and posttest data for subject 3 from the quantitative measurements.

Table 6

*Answers from subject 3 on the qualitative questionnaire, translated into English.*

Table 7

*Raw data on the measurements of BEVS, AAQ-II, SWLS, DASS-21-d, DASS-21-a and DASS-21-s for subject 3.*

|  | **Pretest** | **Posttest** |
| --- | --- | --- |
| **BEVS (total)** | 23 | 21 |
| **AAQ-II** | 54 | 57 |
| **SWLS** | 24 | 25 |
| **DASS-21 - d** | 1 | 1 |
| **DASS-21 - a** | 1 | 1 |
| **DASS-21 - s** | 2 | 6 |

*Note.* N = 1; BEVS = Bull’s Eye Value Survey; AAQ-II = Acceptance and Action Questionnaire II; SWLS = Satisfaction With Life Scale; DASS-d =Depressions subscale in Depression, Anxiety and Stress Scale; DASS-a =Anxiety subscale in Depression, Anxiety and Stress Scale; DASS-s = Stress subscale in Depression, Anxiety and Stress Scale.

*Subject 4.* Subject 4 is a 33-year-old man. He reports that he has not used the application at all during the study. His general opinion is that the startup phase was too demanding, which made him only install the application. He reports that he might have needed encouragement from someone to initiate the usage of the application. The psychoeducation, he reports, was good. The subject accounts for being more aware of his own values and behaviors after doing the exercises in the psychoeducation. Because of the psychoeducation, the subject tried some new behaviors by challenging the thought: “do I practice what I preach?”, he reports. Table 9 shows pre and posttest data for subject 4 from the quantitative measurements.

Table 8

*Answers from subject 4 on the qualitative questionnaire, translated into English.*

Table 9

*Raw data on the measurements of BEVS, AAQ-II, SWLS, DASS-21-d, DASS-21-a and DASS-21-s for subject 4.*

|  | **Pretest** | **Posttest** |
| --- | --- | --- |
| **BEVS (total)** | 21 | 20 |
| **AAQ-II** | 55 | 55 |
| **SWLS** | 27 | 26 |
| **DASS-21 - d** | 2 | 4 |
| **DASS-21 - a** | 1 | 1 |
| **DASS-21 - s** | 6 | 6 |

*Note.* N = 1; BEVS = Bull’s Eye Value Survey; AAQ-II = Acceptance and Action Questionnaire II; SWLS = Satisfaction With Life Scale; DASS-d =Depressions subscale in Depression, Anxiety and Stress Scale; DASS-a =Anxiety subscale in Depression, Anxiety and Stress Scale; DASS-s = Stress subscale in Depression, Anxiety and Stress Scale.

*Subject 5.* Subject 5 is a 37-year-old woman. She reports that she has been using the application almost every day during the study. Also, she reports that she started the application mostly when she was waiting for the bus, as well as when she was being at home. Her general experience of the application is that the idea and concept is great. The psychoeducation, she reports, was detailed, informative and good. The subject accounts for being more aware of her own values and behaviors after starting to use the application, as well as trying to complete new behaviors. She reports that the application gave her incitement to accomplish small things, which made her complete bigger and bigger things. In addition, the application made her starting to reflect over herself, she reports. Table 11 shows pre and posttest data for subject 5 from the quantitative measurements.

Table 10

*Answers from subject 5 on the qualitative questionnaire, translated into English.*

Table 11

*Raw data on the measurements of BEVS, AAQ-II, SWLS, DASS-21-d, DASS-21-a and DASS-21-s for subject 5.*

|  | **Pretest** | **Posttest** |
| --- | --- | --- |
| **BEVS (total)** | 18 | 20 |
| **AAQ-II** | 37 | 43 |
| **SWLS** | 24 | 15 |
| **DASS-21 - d** | 2 | 2 |
| **DASS-21 - a** | 0 | 1 |
| **DASS-21 - s** | 11 | 10 |

*Note.* N = 1; BEVS = Bull’s Eye Value Survey; AAQ-II = Acceptance and Action Questionnaire II; SWLS = Satisfaction With Life Scale; DASS-d =Depressions subscale in Depression, Anxiety and Stress Scale; DASS-a =Anxiety subscale in Depression, Anxiety and Stress Scale; DASS-s = Stress subscale in Depression, Anxiety and Stress Scale.

*Subject 6.* Subject 6 is a 28-year-old man. He reports that he has used the application approximately once a day during the study. Also, he reports that he started the application mostly when he was sitting on the metro. His general opinion of the application is that the structure is good which gave him incitement to use the application often. He reports that, whenever a behavior from the database was completed and checked off in the application, he felt delighted. The psychoeducation, he reports, was very good and pedagogic. The subject accounts for being more aware of his own values and behaviors after starting to use the application. He reports that operationalizing behaviors in the way one has to do with the application made it easier for him to think more of his own behaviors. Also, just seeing the icon of the application in his Iphone, made him think of his own values more. He reports that carrying around Iphone with The application installed is like carrying around your values. Table 13 shows pre and posttest data for subject 6 from the quantitative measurements.

Table 12

*Answers from subject 6 on the qualitative questionnaire, translated into English.*

Table 13

*Raw data on the measurements of BEVS, AAQ-II, SWLS, DASS-21-d, DASS-21-a and DASS-21-s for subject 6.*

|  | **Pretest** | **Posttest** |
| --- | --- | --- |
| **BEVS - total** | 13 | 16 |
| **AAQ-II** | 58 | 60 |
| **SWLS** | 26 | 23 |
| **DASS-21 - d** | 5 | 5 |
| **DASS-21 - a** | 0 | 0 |
| **DASS-21 - s** | 11 | 7 |

*Note.* N = 1; BEVS = Bull’s Eye Value Survey; AAQ-II = Acceptance and Action Questionnaire II; SWLS = Satisfaction With Life Scale; DASS-d =Depressions subscale in Depression, Anxiety and Stress Scale; DASS-a =Anxiety subscale in Depression, Anxiety and Stress Scale; DASS-s = Stress subscale in Depression, Anxiety and Stress Scale.

*Subject 7.* Subject 7 is a 24-year-old woman. She reports that she has used the application a couple of times every day during the study. Also, she reports that she started The application mostly whenever she had accomplished a behavior that was specified in her The application, as well as when she spontaneously came up with a new value or behavior – “the great thing with The application is that it is easy to be spontaneous, carrying around an intervention in the phone makes it easy to use it wherever you are”, she reports. Her general opinion is that the statistics function in the application (described in *Method*) made her feel satisfied. Knowing what behaviors have been accomplished made her feel pleased by her own achievements. Also, she reports that the application motivated her to do things that otherwise would have been postponed or left off. The psychoeducation, she reports, was pedagogic and rich in content, although she experienced it to be a little bit too long. The subject accounts for being more aware of her own values and behaviors after starting to use the application, as well as trying to accomplish new behaviors. She reports that the application gave her a concrete tool to actively work with her values. Also, she reports that simply seeing the icon of the application on the phone made her think of her values and behaviors more. Table 15 shows pre and posttest data for subject 7 from the quantitative measurements.

Table 14

*Answers from subject 7 on the qualitative questionnaire, translated into English.*

Table 15

*Raw data on the measurements of BEVS, AAQ-II, SWLS, DASS-21-d, DASS-21-a and DASS-21-s for subject 7.*

|  | **Pretest** | **Posttest** |
| --- | --- | --- |
| **BEVS (total)** | 19 | 21 |
| **AAQ-II** | 59 | 57 |
| **SWLS** | 24 | 30 |
| **DASS-21 - d** | 2 | 2 |
| **DASS-21 - a** | 0 | 0 |
| **DASS-21 - s** | 5 | 1 |

*Note.* N = 1; BEVS = Bull’s Eye Value Survey; AAQ-II = Acceptance and Action Questionnaire II; SWLS = Satisfaction With Life Scale; DASS-d =Depressions subscale in Depression, Anxiety and Stress Scale; DASS-a =Anxiety subscale in Depression, Anxiety and Stress Scale; DASS-s = Stress subscale in Depression, Anxiety and Stress Scale.

*Subject 8.* Subject 8 is a 25-year-old woman. She reports that she has used the application almost every day during the study. Also, she reports that she started the application mostly when she was waiting for the bus, as well as sitting on the bus. Her general opinion is that the intervention was great for her. She reports that the application was a contributive factor that she started to activate herself, which led to a reduction in depressive symptoms. The psychoeducation, she reports, was very good and pedagogic. The subject accounts for being more aware of her own values and behaviors after starting to use the application, as well as trying to complete new behaviors. She reports that the application started a process where she began to reflect over her own values. Also, after starting to use the application, she feels more satisfied with herself than before, she reports. Table 17 shows pre and posttest data for subject 8 from the quantitative measurements.

Table 16

*Answers from subject 8 on the qualitative questionnaire, translated into English.*

Table 17

*Raw data on the measurements of BEVS, AAQ-II, SWLS, DASS-21-d, DASS-21-a and DASS-21-s for subject 8.*

|  | **Pretest** | **Posttest** |
| --- | --- | --- |
| **BEVS (total)** | 13 | 20 |
| **AAQ-II** | 28 | 40 |
| **SWLS** | 16 | 19 |
| **DASS-21 - d** | 21 | 7 |
| **DASS-21 - a** | 19 | 8 |
| **DASS-21 - s** | 11 | 7 |

*Note.* N = 1; BEVS = Bull’s Eye Value Survey; AAQ-II = Acceptance and Action Questionnaire II; SWLS = Satisfaction With Life Scale; DASS-d =Depressions subscale in Depression, Anxiety and Stress Scale; DASS-a =Anxiety subscale in Depression, Anxiety and Stress Scale; DASS-s = Stress subscale in Depression, Anxiety and Stress Scale.

*Subject 9.* Subject 9 is a 29-year-old man. He reports that he has used the application a couple of times a week during the study. Also, he reports that he started the application mostly when he was sitting on the bus, and on the metro, as well as walking to and from work. His general opinion is that the basic idea of the application is great. The psychoeducation, he reports, was good, although a little bit too long. The subject accounts for being more aware of his own values and behaviors after starting to use the application, as well as trying out new behaviors. He reports that, even though he did not register all behaviors, the application made him start thinking of how he wanted to act, which made him do things that was in line with his values. Table 19 shows pre and posttest data for subject 9 from the quantitative measurements.

Table 18

*Answers from subject 9 on the qualitative questionnaire, translated into English.*

Table 19

*Raw data on the measurements of BEVS, AAQ-II, SWLS, DASS-21-d, DASS-21-a and DASS-21-s for subject 9.*

|  | **Pretest** | **Posttest** |
| --- | --- | --- |
| **BEVS (total)** | 20 | 24 |
| **AAQ-II** | 61 | 63 |
| **SWLS** | 32 | 30 |
| **DASS-21 - d** | 0 | 1 |
| **DASS-21 - a** | 1 | 0 |
| **DASS-21 - s** | 0 | 2 |

*Note.* N = 1; BEVS = Bull’s Eye Value Survey; AAQ-II = Acceptance and Action Questionnaire II; SWLS = Satisfaction With Life Scale; DASS-d =Depressions subscale in Depression, Anxiety and Stress Scale; DASS-a =Anxiety subscale in Depression, Anxiety and Stress Scale; DASS-s = Stress subscale in Depression, Anxiety and Stress Scale.

*Subject 10.* Subject 10 is a 22-year-old He reports that he has used the application two times a week during the study. Also, he reports that he started the application mostly when he was sitting on the tram. His general experience of the application is that it was easy to use and understand. He reports that the open database where users can inspire each other is a great idea, although it requires an amount of users before it will be able to fill its function. More suggestions of behaviors would have made him use the application more, he reports. The psychoeducation, he reports, was pedagogic, although a little bit too long. The subject accounts for being more aware of his own values after starting to use the application. Table 21 shows pre and posttest data for subject 10 from the quantitative measurements.

Table 20

*Answers from subject 10 on the qualitative questionnaire, translated into English.*

Table 21

*Raw data on the measurements of BEVS, AAQ-II, SWLS, DASS-21-d, DASS-21-a and DASS-21-s for subject 10.*

|  | **Pretest** | **Posttest** |
| --- | --- | --- |
| **BEVS (total)** | 20 | 23 |
| **AAQ-II** | 49 | 61 |
| **SWLS** | 27 | 27 |
| **DASS-21 - d** | 1 | 0 |
| **DASS-21 - a** | 0 | 1 |
| **DASS-21 - s** | 3 | 8 |

*Note.* N = 1; BEVS = Bull’s Eye Value Survey; AAQ-II = Acceptance and Action Questionnaire II; SWLS = Satisfaction With Life Scale; DASS-d =Depressions subscale in Depression, Anxiety and Stress Scale; DASS-a =Anxiety subscale in Depression, Anxiety and Stress Scale; DASS-s = Stress subscale in Depression, Anxiety and Stress Scale.

*Subject 11.* Subject 11 is a 30-year-old man. He reports that he has used the application a couple of times a week during the study. Also, he reports that he started the application mostly before going to bed. His general opinion is that it was hard to motivate himself to use the application. He reports that he experienced difficulties with breaking down values to concrete behaviors. Often, the behaviors that he registered were too vague and abstract to be made at once, he reports. Also, the subject reports that he had difficulties with overlooking the open database with other users’ values and behaviors. The psychoeducation, he reports, was good, and that the videos made the education more pleasurable. Subject 11 accounts for being more aware of his own values after the psychoeducation. He also reports that he was more aware of his own behaviors after starting the application. Table 23 shows pre and posttest data for subject 11 from the quantitative measurements.

Table 22

*Answers from subject 11 on the qualitative questionnaire, translated into English.*

Table 23

*Raw data on the measurements of BEVS, AAQ-II, SWLS, DASS-21-d, DASS-21-a and DASS-21-s for subject 11.*

|  | **Pretest** | **Posttest** |
| --- | --- | --- |
| **BEVS (total)** | 16 | 16 |
| **AAQ-II** | 47 | 51 |
| **SWLS** | 15 | 17 |
| **DASS-21 - d** | 2 | 3 |
| **DASS-21 - a** | 3 | 2 |
| **DASS-21 - s** | 4 | 5 |

*Note.* N = 1; BEVS = Bull’s Eye Value Survey; AAQ-II = Acceptance and Action Questionnaire II; SWLS = Satisfaction With Life Scale; DASS-d =Depressions subscale in Depression, Anxiety and Stress Scale; DASS-a =Anxiety subscale in Depression, Anxiety and Stress Scale; DASS-s = Stress subscale in Depression, Anxiety and Stress Scale.
